# Validity and reliability of the athlete diet index in Turkish athletes

**DOI:** 10.3389/fnut.2025.1743113

**Published:** 2026-01-21

**Authors:** Merve Nur Uçak, Pelin Bilgic, Ebru Öztürk

**Affiliations:** 1 Nutrition and Dietetics Department, Faculty of Health Sciences, Acibadem University, İstanbul, Türkiye; 2 Nutrition and Dietetics Department, Hacettepe University, Ankara, Türkiye; 3 Department of Biostatistics, Hacettepe University, Ankara, Türkiye

**Keywords:** athlete diet index, diet quality, sport nutrition, reliability, validity

## Abstract

**Introduction:**

This study aimed to assess the diet quality of Turkish athletes and confirm the validity and reliability of the Athlete Diet Index (ADI), which is the only index designed to assess diet quality in athletes. The ADI assesses three primary domains: basic athlete nutrition, nutrition for exercise performance, and personal dietary practices.

**Methods:**

The final Turkish version of the ADI, developed through forward–backward translation (*n* = 5 + 2), expert review (*n* = 10), and a pilot study (*n* = 15), was administered to 151 professional athletes. Construct validity was examined by assessing the association between ADI scores and 2-day food record scores. Test–retest reliability was evaluated by readministering the ADI to 44 athletes after 3 weeks

**Results:**

All assessments were conducted face-to-face. The ADI demonstrated strong validity (r = 0.798, *p* < 0.001) and good test–retest reliability (ICC = 0.79, 95% CI). Diet quality did not differ by age, sex, or individual versus team sport (*p* > 0.05); however, athletes competing in weight-category sports had significantly lower diet quality than non-weight-category athletes (*p* = 0.01).

**Conclusion:**

The ADI is valid and reliable tool for evaluating the diet quality of Turkish athletes and can be confidently used in both research and applied sports nutrition settings.

## 1 Introduction

Nutrition is one of the most important factors that affect exercise performance. The inclusion of accurate nutritional interventions and appropriate nutrient intake in training programs can help improve the general health status and performance of athletes (1). Athletes have higher requirements than other individuals owing to their intense training programs. These requirements are not constant but instead vary as athletes train at different types, intensities, and durations during the competition period (2).

Athletes need to have high diet quality to minimize nutrient deficiencies caused by inadequate nutrient intake, improve health, and maximize performance by increasing exercise compliance (3).

Diet quality is a parameter that shows how well individuals meet their daily needs. Numerous factors, such as the amount of food consumed, source, hygiene, as well as psychological, sociological, and economic factors, affect diet quality (4).

Diet quality assessments are routinely conducted by nutritionists to determine whether athletes are achieving their health and performance goals. However, many factors make it difficult to assess diet quality in athletes, such as the different training times and intensities among athletes, large portion sizes due to high requirements, and widespread use of supplements (5).

Many diet quality assessment tools have been developed from 1994 to date. Furthermore, there are numerous scales that measure the amount of consumed nutrients and/or food groups, such as the Healthy Eating Index (HEI), Diet Quality Index (DQI), Mediterranean Diet Quality Index (MDQI), and Australian Recommended Food Score (ARFS), and scales that determine the variety of consumed foods (6). Many studies have employed the aforementioned scales to evaluate the diet quality of athletes, some of which are summarized in Table 1.

**Table 1:** Studies that evaluated diet quality in athletes using different scales.

Reference	Group	Scale	Conclusions
Jürgensen et al. (7)	72 Team players (37F) Age: 18.2 ± 2.9 years	HEI-R	According to the scores, there was no athlete belonging to the healthy category. Moreover, 51.4% of the female and 45.7% of the male athletes were found to be “malnourished.” Due to the low correlation between the parameters used in study, investigation of the other parameters affecting the diet quality of athletes was suggested.
Spronk et al. (8)	101 Athletes (64F) Age: 18.6 ± 4.6 years	ARFS	Female athletes had higher diet quality than male athletes (*p* = 0.17). Diet quality did not change according to age, educational level, or sport type. There was a low but positive correlation between nutrition knowledge and diet quality (*r* = 0.252, *p* = 0.024).
Tsoufi et al. (9)	15 Elite basketball players (15M) Age: 24 ± 4 years	HEI	All participants had “enough” diet quality in both race and training days (HEI score > 80). Diet quality was found to be higher on race days than on training days (*p* < 0.001).
Zanella et al. (10)	18 Volleyball players (V) (9F) and 15 Nonathletes (NA) (6F) Adolescents	HEI	While 72.7% of the participants had a low quality diet, none had a good-quality diet. Scores: 43.3 ± 8.2 (V) and 46.4 ± 11.8 (NA) Although the intake of vitamins A and E was lower than recommended, no significant association was observed between diet quality and oxidative stress parameters.
Joaquim et al. (11)	28 Paralympic athletes (7F) Age: 24.9 ± 6.2 years	HEI-R	All athletes are in the “needs regulating” category according to their HEI-R score. Scores: 63.7 ± 5.9 (F) and 61.3 ± 5.3 (M) (*p* > 0.05)
del Mar Fernández-Álvarez et al. (12)	303 Football players Age: 14.15 ± 1.1 years	KidMed	54.8 and 8.9% of the participants showed moderate and low adherence to the Mediterranean diet, respectively. Young people between 13 and 16 years of age are at risk for an obesogenic environment, even if they have adequate levels of physical activity.
Capling et al. (13)	165 Elite athletes (112F) Age: 20 ± 5 years	ADI	There is no significant difference in diet quality according to age or sex. Team athletes have higher diet quality than individual athletes (*p* < 0.05). ADI score: 91.4 ± 12.2
Werner et al. (14)	94 Athletes (73F) Age: 19.9 ± 1.2 years	HEI	Mean HEI score: 59.2 ± 16.6 (low quality) (only 9 athletes had scores ≥80) There was no significant association between diet quality and age, sex, educational level, sport type, and previous nutrition education.
Beba et al. (15)	198 Footballers and Referees (86F) Age: 29.36 ± 8.1 years	HEI-2015	Mean HEI score: 65.04 ± 8.1 (acceptable level) There is a significant association between diet quality and body composition in male athletes and referees but not in female athletes (*p* > 0.05).

F: Female, M: Male.

The aforementioned scales were created considering the general population, and sport-specific factors were not evaluated. Thus, the development of valid and applicable dietary assessment methods that consider the factors that may affect the nutritional status of athletes instead of the general population is recommended (5, 7).

Therefore, Capling et al. developed the Athlete Diet Index (ADI) in 2019 (3) based on the ADI questions, which were mentioned in a master’s thesis in New Zealand in 2016 (8). The ADI evaluates different parameters, such as the diet content and variety, timing of food consumption and hydration according to the exercise program, eating habits (e.g., food preparation and cooking), and use of supplements (8).

To develop the necessary nutritional interventions for improving the performance of athletes, an accurate assessment must first be conducted. The ADI is known to be the first scale that evaluates the diet quality of athletes. It is very important to support and maintain the interventions in all aspects, particularly in the physiological and psychological aspects.

## 2 Methods

### 2.1 Participants

A total of 136 athletes (58 women and 78 men from 15 types of sports) participated in this study. They were older than 16 years (mean age: 19.7 ± 4 years) and have been licensed athletes for at least 2 years. They do not follow any disease-related dietary patterns and have not had any injuries in the last month that could prevent them from training or participating in competitions. The body weights were 61.7 ± 12.8 and 75.1 ± 15.2 kg and the heights were 166.5 ± 9.3 and 176.7 ± 10.5 cm for the females and males, respectively.

### 2.2 Procedure

Permission was obtained from the scientist who developed the scale. This study was approved by the Hacettepe University Noninvasive Clinical Research Ethics Committee (protocol number: 16969557–1658) and the Republic of Turkiye Ministry of Youth and Sports, General Directorate of Education, Research, and Coordination, and Ministry of Sports (protocol number: E-36592570-604.02-4777674).

**Figure 1:**
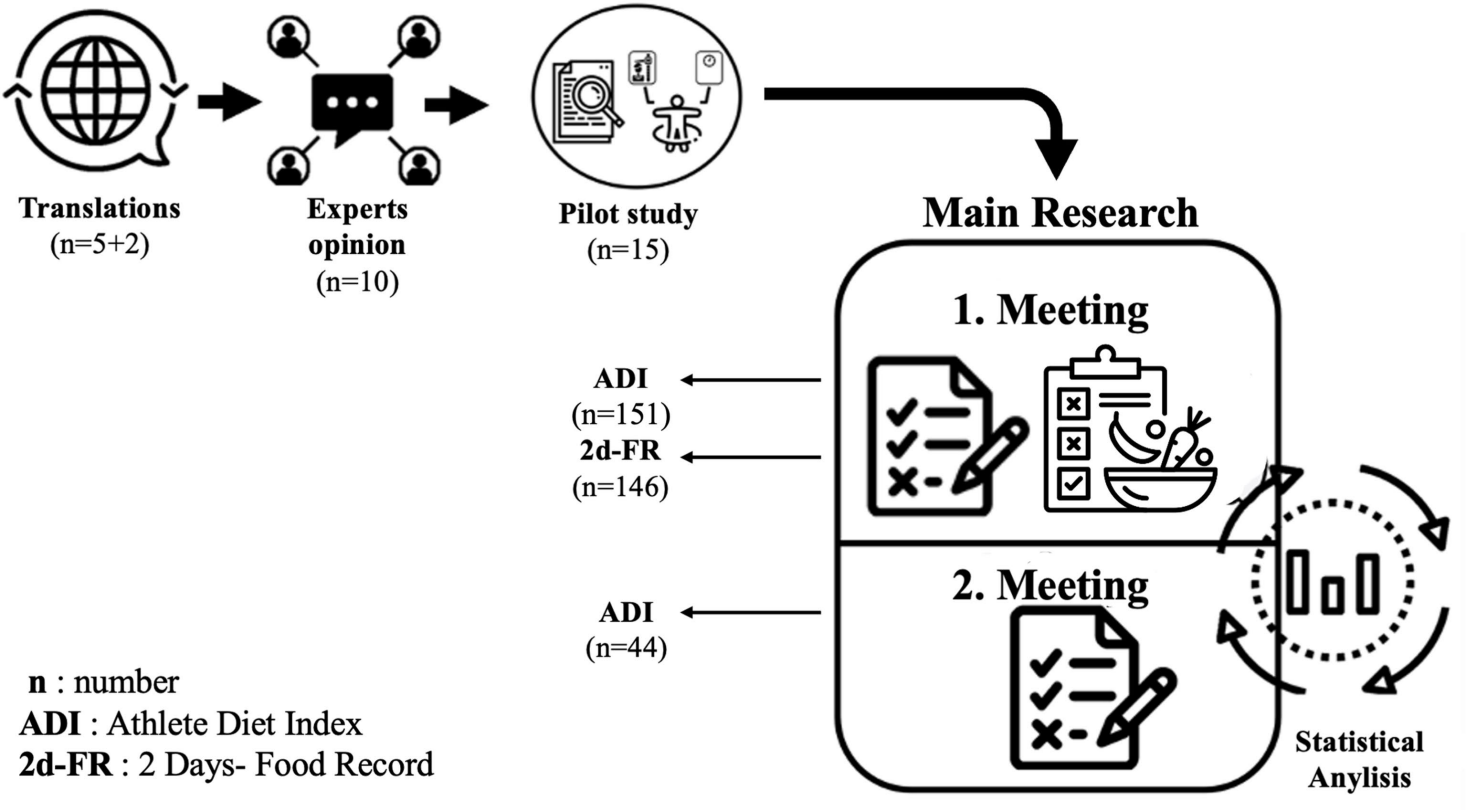
Research design. Step 1: Obtaining permission from the original developers of the ADI and ethical approvals. Step 2: Translation of the ADI into Turkish by five researchers and back-translation into English by two independent researchers. Step 3: Expert panel review by 10 dietitians working in clinical practice and academia to adapt food items to the Turkish context. Step 4: Pilot testing of the adapted ADI in 15 athletes to assess clarity, applicability, and face validity. Step 5: Main study – validity analysis in 136 athletes: administration of ADI-1, collection of 2-day food records (2d-FR), calculation of ADI-FR scores, and correlation analyses. Step 6: Reliability analysis in a subsample of 44 athletes: re-administration of the ADI after 3 weeks (ADI-2), and assessment of test–retest reliability using ICC and Bland–Altman analysis. Step 7: Assessment of diet quality and subgroup comparisons based on ADI-1 scores.

The ADI was translated into Turkish by five researchers and then into English by two researchers who had never seen the original version to confirm the language validity of the scale. The translation and cultural adaptation procedures followed internationally accepted guidelines for cross-cultural instrument adaptation. After the forward translations, the research team performed a reconciliation step to resolve discrepancies and produce a unified Turkish version. A blinded bilingual translator then performed a back-translation into English. The back-translated version was compared with the original ADI to ensure semantic and conceptual equivalence, and discrepancies were resolved through consensus (Figure 1).

During the adaptation of the scale to Turkiye, expert opinions were obtained from 10 dietitians working in both clinical practice and academia. Their feedback guided the removal of food items not commonly consumed in Turkiye and the addition of culturally appropriate alternatives. The expert panel additionally evaluated the cultural relevance of food items and dietary habits to ensure that terminology, examples, and portion references were appropriate for Turkish athletes.

To evaluate the applicability of the final version of the ADI to Turkish athletes and to confirm its face validity, a pilot study involving 15 athletes was conducted. During the pilot phase, comprehension was assessed informally; athletes did not report any difficulties in understanding the items, and only minor wording adjustments were necessary.

In this study, the athletes were interviewed twice. In the first interview, the ADI was administered in Turkish—the culturally adapted version—together with a 2-day food record (2d-FR) to confirm its validity. To confirm reliability, the ADI was readministered 3 weeks later under the same conditions, using a test–retest procedure. Only the Turkish version of the questionnaire was used, including for bilingual athletes, to ensure consistency in data collection. The validity and reliability of the ADI were assessed by examining the association between the scores. A 2-day food record was selected instead of a food frequency questionnaire because the ADI requires detailed day-specific information—including portion sizes, pre- and post-exercise intake, hydration behaviors, and meal patterns—which cannot be accurately assessed using an FFQ.

### 2.3 Athlete diet index

The Athlete Diet Index (ADI) is a sport-specific diet quality questionnaire developed by Capling et al. (3). It consists of three sections: A–Basic Athlete Nutrition (i.e., fruits, vegetables, grains, dairy and alternatives, meat and other alternatives, discretionary foods and alcohol consumption, as well as eating habits, cooking skills, etc.); B –Nutrition for Exercise Performance (i.e., training schedule, nutrition and hydration status at the training program, and use of ergogenic aids); and C – Personal Information (i.e., sex, age, main sport, race level, and anthropometric measurements); it has 68 items in total (3). The ADI is an online scale based on the questions formulated by Rachel Blair (8), a student of the Department of Nutrition and Dietetics at Maassey University, New Zealand, in her master’s thesis titled “Development, Validity and Reliability of the Athlete Diet Index Questionnaire to Assess the Nutrient Consumption Status of High Performance Athletes” (3). The validity and reliability of the ADI were evaluated by the study group that developed the ADI in 2021 (9).

#### 2.3.1 Scoring matrix

Scoring of the ADI was performed by the group that developed the scale (9, 10). The participants received a score based on daily or weekly consumption amounts obtained from the questions and criteria, such as regular food consumption throughout the day in line with the recommendations or pre−/post-exercise fluid and nutrient consumption status. The scoring criteria and cutoff points of the scale were determined by Capling and her team according to the Australian Healthy Eating Guidelines (11) and international sports nutrition recommendations (2). The ADI scores were defined as follows:

- ≥90 points = high-quality nutrition
- 66–89 points = medium-quality nutrition
- ≤65 points = low-quality nutrition

The scoring criteria are summarized in Table A1.

### 2.4 2d-FR

As the ADI also considers meal skipping, eating out, and avoiding certain food groups in addition to the amount of food consumed, it was decided that the most appropriate standard method for comparison was the food record (FR). To determine whether the ADI can accurately measure diet quality, the FRs of the athletes were taken on a normal training day and a rest day for a total of 2 days for comparison. As training-specific nutrition and hydration status were also asked in the ADI, the athletes were asked to record the training time and duration as well as their fluid consumption before, during, and after training on these 2 days. It was also asked where the athletes consumed their meals (home, institutional kitchen, or café). The Food and Nutrition Photo Catalog was used to determine the quantities of food consumed (12). As Turkish cuisine is extremely multifold, the content and quantities of the meals eaten were recorded by taking recipes directly from the athletes or according to the Standard Recipe Book (13). The foods consumed were analyzed using the Nutrition Information System (BEBIS) 8, with adjustments made for any plate waste (i.e., the portion of the food that remained uneaten) (14, 15). The 2-day food record included one weekday and one weekend day in order to capture variation in dietary intake. Standardized written and verbal instructions were provided by a dietitian prior to data collection. Athletes were instructed to use a validated photographic food atlas and a household measures guide to estimate portion sizes. All food records were reviewed and clarified by a registered dietitian during face-to-face meetings to ensure completeness and accuracy.

In addition to analyzing the quantities obtained from the records in the BeBis, these quantities were substituted for ADI in proportion to daily and weekly consumption to obtain an ADI score (ADI-FR) so as to assess the daily macro- and micronutrient intake (9). The nutrients in the FR were divided into the main food groups in the ADI and calculated by proportioning them to the portion amounts given in the ADI score according to the specified consumption amounts. The amounts of all food groups consumed were separately summed and divided by 2, and daily servings were determined. This two-step conversion was necessary due to the prevalence of mixed dishes in Turkish cuisine. Nutrient breakdown enabled precise allocation of components into ADI food groups, which could not be achieved through direct categorization without introducing greater misclassification bias.

### 2.5 Statistical analysis

Statistical analysis was conducted using the IBM Statistical Package for Social Sciences version 23.0 (16). Normality of continuous variables was assessed using the Shapiro–Wilk test, and the distributional characteristics were used to guide the choice between parametric and non-parametric analyses.

For the validity analyses, construct validity was assessed using correlation coefficients between the ADI and 2-day food record variables. Pearson’s correlation coefficient was used when assumptions of normality and linearity were met; otherwise, Spearman’s rank correlation coefficient was applied. A correlation between 0.5 and 0.7 indicates that the measurement tools assess a similar construct and provides sufficient evidence for convergent validity (17, 18).

For the reliability analyses, test–retest reproducibility of the ADI was evaluated using the intraclass correlation coefficient (ICC). A two-way random-effects model with absolute agreement (ICC(2,1)) was employed, which is recommended for assessing consistency of repeated measurements in independent samples. Prior to ICC computation, assumptions of normality (Shapiro–Wilk), homogeneity of variances, and the absence of influential outliers were examined and satisfied. ICC values were interpreted as follows: <0.50 = poor reliability; 0.50–0.75 = moderate reliability; 0.75–0.90 = good reliability; and >0.90 = excellent reliability (18).

Agreement between test and retest scores was further examined using Bland–Altman analysis. Bland–Altman plots were generated using the “BlandAltmanLeh” package (18) in R software version 4.3.0 (19), and mean differences with 95% limits of agreement were calculated.

## 3 Results

Of the 151 athletes who participated in the study, 10 dropped out halfway and 5 did not provide the 2-day food record (2d-FR), although they completed the ADI. Consequently, the data of the remaining 136 athletes were analyzed. The data of 44 athletes who agreed to be re-interviewed were included in the reliability analysis. Among the participants, 94% were individual athletes and 34.6% were weight-class athletes.

Athletes represented 15 different sports, including both weight-bearing (e.g., athletics, basketball, combat sports) and non–weight-bearing disciplines (e.g., swimming, rowing, cycling). Weight-bearing classification was based on whether the athlete supported their own body weight against gravity during impact-loading movements. The majority of the athletes competed at the national or international level and 64% trained for more than 16 h per week. Male and female athletes were similarly represented across sport types, with no major imbalance between weight-bearing and non–weight-bearing categories.

A comparison of the validity sample (*n* = 136) and the reliability subsample (*n* = 44) showed no meaningful differences in age, training hours per week, sex distribution, or distribution of sport types, indicating that both samples were comparable in their demographic and sport-related characteristics.

### 3.1 Dietary analysis from the 2d-FR

The mean daily energy intake of the athletes was 2,385 kcal (females: 2038 kcal; males: 2630 kcal), and the mean fluid intake was 3,769 mL (6 athletes consumed less than 2 L of fluid). The mean daily carbohydrate, protein, and fat intakes were 239 g (3.54 g/kg), 107.3 g (1.59 g/kg), and 108.9 g (41% of the total energy), respectively. Moreover, the mean daily intakes of iron and calcium — the two micronutrients specifically assessed within the ADI scoring system — were 13.3 mg (females: 11.7 mg; males: 14.4 mg) and 846 mg (<18 years: 747 mg; 19–50 years: 947 mg), respectively. The nutrient intakes did not significantly differ between the groups according to sex or age (*p* > 0.05) (Table 2).

**Table 2:** Mean energy and nutrient intake obtained from 2d-FR.

Variable	Mean	SD	Min	Max
Energy (kcal)	2,385	803.8	898	5,678
Female	2038	678	898	4,549
Male	2,630	798	1,351	5,678
Water (mL)	3,769	1119.7	500	8,600
Carbohydrate (g)	239	93.8	53.2	601
Carbohydrate (g/kg)	3.54	1.37	0.84	8.24
Protein (g)	107.3	36.4	36.7	220.4
Protein (g/kg)	1.59	0.5	0.5	3.13.1
Fat (g)	108.9	40.2	34.3	260.8
% Fat energy	41.1	5.8	21.9	57
Fiber (g)	19.8	7.4	7.5	50
Iron (mg)	13.3	4.9	5.4	30.2
Female	11.7	5.2	5.4	26.4
Male	14.4	4.2	6.1	30.2
Calcium (mg)	846	736.8	162	8,356
<18 years	747	303.4	162	1,489
19–50 years	947	1,001	322	8,356

SD: Standard deviation, min: minimum, max: maximum.

Based on the intake values obtained from the 2d-FR, we examined whether individuals met their age- and sex-specific nutritional requirements (20). With these values, it was concluded that half and a quarter of the athletes could not meet their calcium and iron intake requirements, respectively (Table 3).

**Table 3:** Evaluation of athletes’ nutrient intake status according to RDA levels (percentage of amounts met).

Nutrient	% RDA					
	<%67		%67–133		>%133	
	*n*	%	*n*	%	*n*	%
Calcium	71	52.2	57	41.9	8	5.9
Iron	33	24.3	65	47.8	38	27.9
Vitamin A	23	16.9	50	36.8	63	46.3
Vitamin E	7	5.1	31	22.8	98	72.1
Vitamin B1	47	34.6	77	56.6	12	8.8
Vitamin B2	9	6.6	71	52.2	56	41.2
Vitamin B6	23	16.9	70	52.5	43	31.6
Folate	81	59.6	48	35.3	7	5.1
Vitamin C	48	35.3	51	37.5	37	27.2
Potassium	101	74.3	34	25	1	0.7
Magnesium	53	39	77	56.6	6	4.4
Phosphorus	8	5.9	60	44.1	68	50

### 3.2 Validity of the athlete diet index

The language validity of the ADI was established using translation (*n* = 5) and backtranslation (*n* = 2) methods, whereas the face validity was confirmed through a pilot study (*n* = 15).

Construct validity was assessed by convergent validity between the ADI-1 and ADI-FR scores (21). According to the total score, the validity of the ADI was confirmed (*r*s > 0.7). When the subscores were analyzed, it was found that these groups measure similar constructs with *r*s = 0.795 for Group A and *r* = 0.504 for Group B but these groups are not sufficient to measure similar constructs with *r* = 0.336 for Group C (*p* < 000.1, two-way) (Table 4).

**Table 4:** Comparison of scores derived from ADI-1 and ADI-FR.

Score	ADI version		Correlation coefficient
	Mean	SD	(*r*)
Total score (125)			
ADI-1	67.4	14.7	0.798*
ADI-FR	67.3	12.5	
A: Basic nutrition score (80)			
ADI-1	37.3	9.7	0.795**
ADI-FR	39.5	8.9	
B: Specific nutrient score (35)			
ADI-1	22.1	5.4	0.504*
ADI-FR	19.9	4.4	
C: Nutrition habits score (10)			
ADI-1	8	1.6	0.336*
ADI-FR	7.8	1.8	

SD: Standard Deviation, *p* < 0.001 (two-way). * Spearman correlation coefficient was used. ** Pearson correlation coefficient was used.

### 3.3 Reliability of the athlete diet index

The two face-to-face administrations of the ADI were conducted 3 weeks apart. No difference was observed between the total scores and subscores (except for B: specific nutrient score) (*p* > 0.05). There was a good correlation with *r* = 0.791 in total scores. However, the coefficient decreased as the chapters of the ADI progressed. The Bland–Altman analysis revealed that all but two athletes were within the upper and lower limits, and there was no evidence of systematic bias. Test–retest scores were also examined for potential systematic changes using paired-sample comparisons, and no significant differences were observed between administrations (*p* > 0.05), confirming stability across time (Table 5).

**Table 5:** Comparison of the scores obtained from ADI-1 and ADI-2.

Score	ADI version		Mean difference (95% CI)		
	Mean	SD	Mean (%95 CI)	P	ICC (%95 CI)
Total score (125)					
ADI-1	72.8	13.1	1.19 (−1.89, 2.89) b	0.675*	0.79a (0.65–0.88)b
ADI-2	72.3	10.9			
A: Basic nutrition score (80)					
ADI-1	41.1	9.5	0.99 (−0.11, 3.87) b	0.064*	0.71 a (0.52–0.83) b
ADI-2	39.2	8			
B: Specific nutrient score (35)					
ADI-1	23.3	4	0.50 (−2.08, 0.6) b	0.039*	0.63 a (0.40–0.78) b
ADI-2	24.4	3.9			

	P25	M	P75	*P*	ICC (95% Cl)
C: Nutrition habit score (10)					
ADI-1	7	8	9	0.072**	0.582 a (0.25–0.75) b
ADI-2	8	9	10		

^*^ Measured with the Paired Samples Significance Test. ^**^Measured with the Wilcoxon Signed-Rank Test. a: *p* < 0.001, b: up and low limits, CI: confident interval, ICC: Intra Class Correlation Coefficient.

### 3.4 Assessment of diet quality

To provide a broader data pool, general information about the athletes was created from the data obtained during the initial interview. The participants obtained an average ADI score of 67.4. Although the female athletes (67.88) obtained a higher ADI score than the male athletes (66.97), no significant difference was observed between them (*p* > 0.05). When comparing athletes who competed in weight-category sports with those who did not, the group without weight-category sport participation had a higher ADI score (69.5) than the group with weight-category sport participation (63.3). A significant association was found between participation in weight-category sports and diet quality, with athletes competing in weight-category sports exhibiting lower ADI scores (*p* < 0.05). The lower diet quality observed in weight-category athletes may be related to sport-specific practices such as rapid weight reduction, energy restriction, dehydration strategies, and cyclical dieting commonly used before competitions. These strategies are well documented to negatively affect diet quality and micronutrient adequacy in weight-focused sports (22). Furthermore, diet quality was not found to be associated with age, sport type, and race level (*p* > 0.05) (Table 6).

**Table 6:** Diet quality scores obtained from ADI-1.

Score	*N*	Mean	SD	*p*
Gender				
Male	78	66.97	14.66	0.724^1^
Female	58	67.88	14.93	
Weight class				
Yes	47	63.30	15.23	0.019^1^
No	89	69.51	14.07	

	*N*	Median	P25	P75	*p*
Age (years)	136	18	17	21	0.664^2^
Sport type					
Individual	129	68	56	80	0.969^3^
Team	7	68	65	73	
Race level					
Regional	7	66	46	83	0.579^4^
National	34	68.5	57	76	
International	95	68	57	80	

SD: Standard deviation. 1: T-Test for Equality of Means, (*p* = 0.5, two-way). 2: Spearman correlation coefficient. 3: Mann–Whitney *U* Test (*p* = 0.05, two-way). 4: Kruskal Wallis Test (*p* = 0.05, two-way).

## 4 Discussion

Nutrition is a key factor among the intervenable factors that affect athlete performance. To establish the accurate nutritional interventions for improving the performance of athletes, their nutritional status must first be determined. By evaluating the diet quality, the athletes’ adherence to the requirements and the deficiencies in their diet can be determined, and thus, necessary recommendations can be made accordingly.

In our country, there is a lack of measurement tool that evaluates diet quality specific to athletes. This study aimed to introduce the ADI, which is an unprecedented scale in the international literature, into our literature and use it as a measurement tool to evaluate the diet quality of athletes in future studies. Future research is expected to generate data that could inform culturally and contextually appropriate updates to the scale for Turkish athletes.

The ADI is a scale that athletes can self-administer on an online platform (9). However, based on the observations in the pilot study (*n* = 15), it was suggested that the administration of the scale by a practitioner would yield more accurate results. Therefore, in the main study, the co-researcher asked the questions and the athletes answered. According to the opinions received from the athletes after the administration of the scale, it was concluded that the questions were easy to understand and closely related to sports nutrition.

### 4.1 Validity of the athlete diet index

For construct validity, *r* = 0.5–0.7 between the 2d-FR and ADI scores was sufficient to prove convergent validity (17). A high level of correlation was observed between the data, and the ADI was found to be a valid diet quality measurement tool for Turkish athletes. Capling et al. (9) obtained *r* = 0.69 with the same *p-*value. The correlations observed in this study were higher than those reported in the original validation study, particularly for Sections A and B (9). As the scoring of Section A directly assesses the amounts of consumption and FR, a coefficient value of *r* > 0.7 was expected. For this section, both assessment methods appeared to measure the same constructs. As shown in Table A1 in the “B: Special Nutrients” section, unsaturated fatty acids consumed, types of fruits and vegetables, availability of nuts or whole-grain bread, and consumption of foods rich in calcium and iron minerals were asked. In this study, difficulties were encountered in determining the variety of foods consumed during the whole week with a 2d-FR. In addition, the 2d-FR was inferior to the 4d-FR in explaining the consumption of the whole week, particularly in terms of diversity and availability, as some foods may not have been consumed on these days but have been consumed on other days. The greatest limitation of this section was that the frequency of food consumption for 1 week was not determined in addition to the FR. However, it was concluded that similar structures were evaluated, albeit close to the lower limit set in this section.

Although a 2-day food record was used as the criterion measure in this study, this method inherently captures only short-term intake and may not fully reflect habitual dietary patterns. Longer dietary assessments (e.g., 3–7 day records or food frequency questionnaires) are often recommended for evaluating long-term diet quality. However, the ADI measures day-specific nutritional behaviors—including pre- and post-exercise intake, hydration practices, and meal timing—that cannot be accurately assessed using longer recall-based tools. Therefore, a 2-day record including one training day and one rest day was selected as the most appropriate comparator. Nevertheless, the limited duration of dietary recording may have contributed to underestimating intake variability, which could influence the observed strength of the validity correlations. Future research using longer assessment periods may help further clarify these associations.

The correlation coefficient between ADI-1c and ADI-FRc was insufficient to prove validity. Capling et al. (9) obtained *r* = 0.76 with the same *p-*value. The most important reason for the difference between these two groups was that the 2d-FR was taken by one group whereas the 4d-FR was taken by the other. Part C, which examines dietary habits, considers meal skipping; nutrition before, during, and after exercise; and fluid intake (a total of 6 points). The weak correlation between the two methods (*r* = 0.336) could be attributed to the fact that the assessment was based on data from only 1 day of exercise out of the 2 days recorded. Moreover, the FR is a measurement tool that only assesses food consumption. In this study, nutrition in relation to exercise was directly assessed by the researcher to yield more accurate results. Finally, as the scoring of this section was out of 10, the fact that even small differences in the mean and standard deviation significantly affect correlation cannot be disregarded.

As the chapters progressed, the correlation coefficient between the ADI score and the scores obtained from the FR decreased. This could be attributed to the fact that the content of the chapters progresses in relation to quantity, variety, and habit. Although the quantities obtained with the 2d-FR reflect the amount of consumption, they do not clearly show habits in particular.

The ADI does not only evaluate the amount of consumption (3). Therefore, it is likely that no strong association exists between daily energy intake according to the 2d-FR and ADI scores (*r* = 0.325, *p* < 0.001, bidirectional). Accordingly, a high daily energy intake does not mean that the diet quality is high; conversely, a relatively low daily energy intake does not mean that the diet quality is low. However, it is extremely important to consume adequate amounts of nutrients as a low-energy diet that does not meet the requirements will lead to a decrease in diet quality (5). In this study, although there were athletes whose consumption amounts were below the requirements, some micronutrients were taken in insufficient amounts, although the recommended amounts were generally met. This explains why the participants in this study had moderate-quality nutrition.

Bland–Altman analysis was not used for the validity comparison between the ADI and the 2-day food record because the two tools do not measure the same construct and are not intended to be interchangeable. While the food record quantifies actual intake, the ADI evaluates diet quality through categorical scoring. Therefore, correlation analysis was deemed the most appropriate statistical method for examining convergent validity.

### 4.2 Reliability of the athlete diet index

At the evaluation for the reliability analysis of the ADI, the Bland–Altman analysis for repeated measures revealed a mean difference of 0.5 for the total scores (lower limit: −14.91; upper limit: 15.91) and no evidence of systematic bias in the regression line. The study in which the scale was developed (9) did not find a bias, and the mean difference was 1.9 (lower limit: −17.8; upper limit: 21.7), which was higher than the value obtained in the present study. All but two athletes were within the upper and lower limits. This gap between the upper and lower limits could be attributed to the fact that the scale was scored over 125 or that the dietary intake of the athletes changed daily in the 3 weeks between the two interventions.

No significant difference was observed in the total scores between the two measurements. Furthermore, the reliability of the ADI was good (18). The mean difference obtained by Capling et al. (9) was higher than that obtained in the present study. When the subscores were examined in detail, no significant difference was observed in the total or subscores between the first and second measurements (*p* > 0.05). The mean difference between the measurements for the Special Nutrients score indicated a significant difference for this section (*p* < 0.05). Although similar to the results yielded by Capling et al. (9), the fact that this difference was significant despite the smaller mean difference than the other subscores could be attributed to the lower total score of Section B than Section A or to the change in the consumption of specific nutrients, such as unsaturated fatty acids, antioxidants, calcium, and iron, which was questioned in this section, over the intervening 3-week period.

The ICC values of the sections indicated good and moderate reliability. Capling et al. (9) reported a similarly high correlation for the total score and higher correlations for the subscores than those observed in the present study. The fact that Capling et al. (*n* = 68) worked with approximately 1.5 times as many athletes as in this study (*n* = 44) relatively increased the reliability.

As a result of all these statistics conducted for the assessments, it can be concluded that the scale is well valid and moderately to well reliable. Although radical changes in dietary habits may not occur over a short time period, such as 3 weeks, changes in dietary habits can be observed in athletes depending on the changes in their training programs. The decrease in reliability toward the final sections indicates that the comprehensiveness and length of the scale may cause the participants to lose focus, leading to responses that do not reflect reality. Although basic information on weekly training frequency was collected, detailed training-volume data (e.g., periodized load, session duration, and intensity) were not included in the questionnaire. Therefore, it was not possible to directly verify whether changes in dietary habits were associated with changes in athletes’ training programs.

In addition to the study in which the ADI was developed and validated (9), there are very few studies evaluating the validity of dietary assessment methods specific to athletes. Ward et al. evaluated calcium intake in female athletes (23); they compared the calcium intake control chart they prepared with the 6-day FR (*n* = 34, ICC = 0.41, *p* = 0.0067). The reliability of the scale was evaluated using the test–retest method (*n* = 56, ICC = 0.54, *p* < 0.001). A lower level of reliability than that achieved in this study was observed. Baker et al. (24) reported correlations between daily energy (*r* = 0.52), amounts of carbohydrate (*r* = 0.29) and protein (*r* = 0.61) measured using the 24-h digital diet reminder tool, and the 24-h retrospective food recall. In particular, it has been reported that athletes receiving higher amounts of energy exhibit substantial differences in estimating how much they consume (24). This suggests that larger amounts consumed are more difficult to remember. Accordingly, in this study, the correlation between the reported (ADI) and calculated (ADI-FR) portion sizes was moderate despite the high energy intake of the athletes, which can be considered to be a good result in terms of consistency.

Sunami et al. (25) compared food consumption frequency with the FR in 3 non-consecutive days among university athletes and obtained *r* = 0.30 (−0.08–0.72) for 19 different food groups. This could be attributed to the fact that athletes have difficulty remembering the type and amount of food they consume, which leads to under- or over-reporting because their training programs vary in terms of time and content throughout the day or because they consume large portions to meet their requirements.

**Figure 2:**
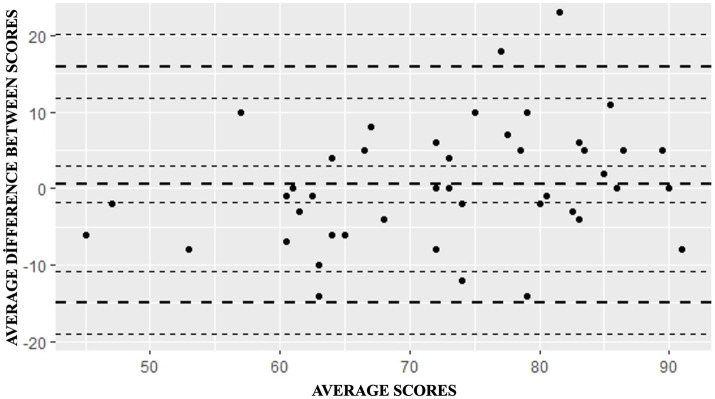
Bland–Altman Plot of the scores obtained in SDI-1 and SDI-2 applications (*n* = 44). The long and relatively thicker dashed lines represent the mean difference between the scores, while the shorter and smaller dashed lines represent the lower and upper levels ± 1.96. Mean difference: 0.5, Lower limit: −14.913 and Upper limit: 15.913, and no systematic bias is observed.

Although methodological differences make it challenging to directly compare this study with other studies—except for the validity and reliability study conducted on the original version of the scale—it is possible to make associations between them according to the results (Figure 2).

### 4.3 Dietary assessments of athletes

The fact that the energy requirements differ according to the type, intensity, and duration of exercise performed during the day explains the large standard deviation in energy intake. Although fluid consumption recommendations are directly associated with the amount of sweating, they are known to vary among athletes. However, in this study, 36 athletes complied with the WHO guidelines, which recommend daily consumption of 4.5 L of fluid for active individuals (26). The large standard deviation in fluid consumption indicates that the differences in the sports branch have varying effects on the required and consumed amounts.

The carbohydrate consumption of 3.5 ± 1.4 g/kg obtained in this study is equivalent to the lower limit according to the recommendations (27). It has been reported that the predominant energy system changes according to the type and intensity of the exercise performed and that the amounts of carbohydrate to be consumed according to the requirements differ from each other (28). The average consumption met the daily protein intake recommendation of 1.2–2 g/kg (29), but 30 athletes did not reach the lower limit. Overall, 61% of the athletes met the lower limit for the recommended carbohydrate intake (30) (3–5 g/kg/day), whereas 77.2% of them met the recommended protein intake (1.2 g/kg/day) (29).

The participants of this study consumed high amounts of fat. However, in addition to the total amount of fat consumed, the type of fat is also extremely important. In this study, the average consumption of unsaturated fatty acids was found to be 61.1 g/day. The remaining amount is known to be saturated fat. The athletes in this study consumed higher amounts of saturated fat. It is well known that animal sources rich in protein also have high saturated fat content (31). This could be associated with the fact that the athletes who participated in the study consumed 1.59 g/kg of protein daily according to their FRs, consumed snacks with high saturated fat content, and frequently preferred full-fat dairy products with high saturated fat content.

The athletes did not meet the recommended daily fiber consumption of 25 g (an average of 19.8 g/day) (32). This suggests that the fiber consumption of the athletes in this study was insufficient. The preference for white bread and the low amount of fruits and vegetables consumed daily explain such an insufficiency.

When the micronutrient intake levels obtained from the FR analysis were compared with the RDA (20) calcium, folate, and potassium intake was generally inadequate in more than 50% of the athletes. This could be attributed to the low consumption of milk and dairy products rich in calcium as well as green leafy vegetables, which are sources of potassium. According to the data obtained, the high levels of vitamin E intake can be explained by the fact that the meals were mostly prepared using sunflower oil. In a review by Jordan et al. that examined micronutrient deficiencies in athletes, studies conducted in different sports disciplines were evaluated; it was found that the intake levels of vitamins E and C were sufficient in general but the consumption of vitamin B12, folate, iron, calcium, and magnesium minerals did not meet the requirements (33).

### 4.4 Assessment of diet quality

The participants of this study were found to have a medium-quality diet with a mean score of 67.4, and this did not significantly differ according to age, sex, level of competition, and whether they were individual or team athletes (*p* > 0.05). A previous study that examined the association between nutritional knowledge and diet quality using ARFS reported that the diet quality of women was higher than that of men; however, there was no significant association between age, educational level, sport type, and diet quality (34). In another study that investigated the diet quality of athletes using HEI, diet quality was not found to have a significant association with sex, sport type, and whether nutrition education was received (*p* > 0.05) (35).

Spronk et al. reported that the diet quality of women was higher than that of men. In this study, age, sex, educational level, and being a team or individual athlete did not significantly change diet quality. Although athletes who received nutrition education had higher nutritional knowledge, no significant change was observed in their diet quality (*p* = 0.466) (34).

In this study, although the diet quality scores of male athletes were lower than those of female athletes (M: 66.97; F: 67.88), the difference was not significant (*p* = 0.724). The mean diet quality score of the weight athletes was 63.3, whereas that of non-weight athletes was 69.5, indicating a significant association between weight and diet quality (*p* < 0.05). These results indicate that the diet quality of weight athletes is lower. To compete in the targeted weight class, these athletes tend to have low-quality nutrition by making various restrictions in their diet programs with wrong practices during the precompetition period.

Although diet quality did not differ significantly by age, sex, or sport type in the present study, several factors may explain these non-significant findings. First, the sample consisted predominantly of national and international-level athletes who are typically exposed to similar nutritional messages, coaching practices, and performance-oriented expectations regardless of demographic characteristics. Homogeneity in competitive level and training demands may therefore reduce between-group variability (36). Additionally, the relatively narrow age range of the sample (mainly late adolescence to young adulthood) may have limited the potential to detect age-related nutritional differences that are more evident across wider age spans. Previous studies using HEI, ARFS, and similar indices have likewise shown minimal differences in diet quality across sex and sport type among elite athletes, suggesting that performance-driven nutritional routines may overshadow demographic influences (37).

The lower diet quality observed in weight-bearing and weight-class athletes may be attributed to sport-specific cultural and physiological factors. Sports such as combat sports, gymnastics, and endurance running are characterized by pressures related to leanness, weight manipulation, and rapid weight cutting. These pressures often result in restrictive eating patterns, meal skipping, dehydration strategies, and monotonic diets that compromise dietary variety and micronutrient adequacy (38). Evidence suggests that weight-focused sports display higher prevalence of low energy availability (LEA) and disordered eating behaviors, both of which are associated with poorer diet quality and reduced micronutrient intake (39). In contrast, non–weight-bearing sports generally emphasize fueling strategies for performance and recovery rather than body weight manipulation, which may support more balanced dietary behaviors. These mechanisms provide plausible explanations for the lower ADI scores found among weight-bearing athletes in this study.

The results of this study indicate that both female and male athletes have a moderate level of quality nutrition. This could be associated with the inadequate intake of micronutrients, such as vitamins B1 and C, folate, potassium, and calcium, due to the insufficient consumption of vegetables and fruits, frequent preference for white bread and full-fat dairy products, frequent skipping of breakfast, and lack of dietary diversity. Using the same scale for different athletes, one study (9) found that athletes had a “moderate-quality” diet (84.1), and another (40) found that Australian athletes had a “high-quality” diet (91.4). In addition, Capling et al. reported that team athletes had a higher diet quality than individual athletes (92.7 vs. 88.5, *p* < 0.05) (40). Team athletes may influence each other’s nutritional behaviors more strongly, whereas individual athletes—who are relatively more isolated—may experience less social influence. However, such influence does not necessarily lead to improved diet quality.

Jürgensen et al., using HEI-R (7), found “needs adjustment” (51.9) in team athletes; Burrows et al., using A-ARFS (41), found “good quality” (34) in rugby athletes; Spronk et al. (34) found “good quality” (33.6) in elite athletes, Tsoufi et al. (42) found 89.7% (out of 100) using the HEI, Martin et al. (43) 65.2 (out of 100) using the HEI-2010, Zanella et al. (44) 43.3% (low quality with <51%) using the HEI, Joaquim et al. (45) M: 61.3% and F: 63.7, Beba et al. (46) recorded a diet quality of 65.4 (out of 100) in soccer players using the HEI-2015. It is difficult to relate these studies with each other due to differences in the parameters and cutoff points used.

## 5 Limitations

In the planning phase of the study, it was initially intended to collect 3-day food records (3d-FR), including one normal training day, one low-intensity training day, and one non-training day. However, due to incomplete submissions during data collection, the assessment was limited to a 2-day food record (2d-FR) consisting of one training day and one rest day. In the original ADI validation study (19), the use of a 4-day food record provided a stronger basis for comparison. Nevertheless, despite using fewer days of dietary recording, this study analyzed a larger number of food records (*n* = 136) than the original study, which may partially compensate for this limitation.

Additionally, several methodological limitations should be acknowledged. First, both the ADI and the 2-day food records relied on self-reported dietary intake, which is subject to recall bias, misreporting, and underestimation—particularly among athletes with variable training schedules. Second, the 2-day dietary assessment may not fully capture habitual intake or weekly variation, potentially affecting the strength of convergent validity. Third, although the test–retest sample size (*n* = 44) is comparable to similar validation studies, it remains modest and may limit the precision of ICC estimates. Future research using longer dietary assessment periods and larger reliability subsamples would help strengthen generalizability and methodological robustness.

## 6 Conclusion

The ADI is the first and only scale designed to evaluate the diet quality of athletes. The ADI score, which captures food intake patterns, training-specific nutrition practices, and habitual dietary behaviors, is positively associated with overall diet quality. With this study, the ADI has been culturally adapted and validated for use among Turkish athletes, making it available for both dietitians in clinical practice and researchers in academia.

This study demonstrated that the ADI provides a practical and informative overview of athletes’ nutritional status; however, several limitations should be acknowledged. First, both the ADI and the 2-day food record rely on self-reported intake, which may introduce recall or reporting bias. Second, the use of only two food record days may not fully capture habitual intake, despite efforts to include one training and one rest day. Third, the test–retest reliability analysis was conducted with a moderate sample size.

Future research should explore additional dimensions of validity, including longitudinal predictive validity, the applicability of the ADI to younger adolescent athletes, and its relationship with performance-related metrics such as training load, recovery, and competition outcomes. Such studies may provide further insight into the scale’s utility and its potential contribution to athlete monitoring systems. Overall, the ADI is a valuable tool for guiding nutritional assessment and intervention strategies aimed at supporting the optimal performance and health of athletes.

## Data Availability

The raw data supporting the conclusions of this article will be made available by the authors, without undue reservation.

## Appendix

**Table A1:** Athlete diet index detailed scoring.

Food group/component	Australian dietary guidelines	Max. score	Criteria for max. score	Criteria for min. score	Scoring cut-points
Core nutrition					
Fruit	*Eat a wide variety of fruit*	10	>3 serves/d	0 serves/d	0 = 0–0.9 serves/d 4 = 1–1.4 serves/d 6 = 1.5–1.9 serves/d 8 = > 2–2.9 serves/d 10 = > 3 serves/d
	Fruit variety	5	> 5 types/week	No fruit	0 = no fruit 1 = 1 type 2 = 2 types 3 = 3 types 4 = 4 types 5 = > 5 types
Vegetables	*Eat a wide* var*iety of vegetables*	10	> 5 serves/d	0 serves/d	0 = 0–0.9 serves/d 1 = 1–1.9 serves/d 2 = 2–2.9 serves/d 5 = 3–3.9 serves/d 8 = 4–4.9 serves/d 10 = > 5 serves/d
	Vegetable variety	5	> 10 types/week	No vegetable	0 = 0 types 1 = 1–2 types 2 = 3–4 types 3 = 5–6 types 4 = 7–9 types 5 = > 10 types
Grains	*Eat a wide variety of grain foods; mostly wholegrain or high fibre*	10	> 6 serves/d	0 serves/d	0 = 0–0.9 serves 1 = 1–1.9 serves 2 = 2–2.9 serves 5 = 3–3.9 serves 7 = 4–4.9 serves 8 = 5–5.9 serves 10 = > 6 serves
	Wholegrain bread	5	Wholegrain	Refined or none	0 = do not eat bread 1 = gluten free bread (low fiber), white bread 3 = wholemeal bread 5 = wholegrain, other bread types (dense/heavy)
Dairy and dairy alternatives	*Eat a wide variety of milk, yoghurt, cheese and/or alternatives*	10	> 3 serves/d	0 serves/d	0 = 0–0.9 serves/d 4 = 1–1.4 serves/d 6 = 1.5–1.9 serves/d 8 = 2–2.9 serves/d 10 = > 3 serves/d
	Mostly reduced fat	5	Skim	None	0 = none 1 = full fat 3 = reduced fat 5 = skim, fat free
Meat and meat alternatives^^^	*Eat a wide variety of lean meat, fish, poultry, eggs, tofu, nuts and seeds, legumes and beans*	10	>3 serves/d	0 serves/d	0 = 0 serves/d 1 = 0.1–0.9 serves/d 2 = 1–1.4 serves/d 5 = 1.5–1.9 serves/d 8 = 2–2.9 serves/d 10 = > 3 serves/d
Discretionary foods^*^	*Limit foods high in saturated fat, salt and added sugar*				
	Processed and deli meats	1	< 2 d/week	> 3 d/week	0 = > 3 days/week 1 = < 2 days/week
	Sweet and savory snack foods#	3	< 1 serve/d	> 4 serves/d	0 = > 4 serves/d 1 = 2.1–3.9 serves/d 2 = 1.1–2 serves/d 3 = < 1 serves/d
	Takeaway/fast food	3	< 1 d/week or ‘rarely’	> 4 d/week	0 = > 4 days/week 1 = 3 days/week 2 = 2 days/week 3 = < 1 day/week or rarely
Alcohol^*^	*Limit alcohol intake*	1	< 2 d/week or ‘rarely’	> 3 d/week	0 = > 3 days/wk. 1 = < 2 day/wk. or ‘rarely’
	Binge drinking	2	< 4 serves/week	> 8 serves/week	0 = > 8 serves/wk. 1 = 5–7 serves/wk. 2 = < 4 serves/wk
Sub-score		80			
Special nutrients					
Essential fats	Regular intake of unsaturated fats (e.g., nuts/seeds, fish, oils)	5	>5 types/week	None	0 = none 1 = 1 type 2 = 2 types 3 = 3 types 4 = 4 types 5 = 5 types
Antioxidants^+^	Frequency of fruit intake	1	>5 d/week	< 4 d/week	0 = < 4 days/wk. 1 = 5 days or more
	Frequency of vegetable intake	1	>5 d/week	< 4 d/week	0 = < 4 days/week 1 = 5 days or more
	Variety fruit	3	>3 specific types/week	None	0 = none 1 = 1 type 2 = 2 types 3 = ≥3 types or more
	Variety vegetables	3	> 3 specific types/week	None	0 = none 1 = 1 type 2 = 2 types 3 = 3 types
	Nuts and seeds	1	Consume nuts/seeds	Not consume	0 = does not eat 1 = eats
	Grains (mostly wholegrain)	1	Mostly wholegrain	Other types bread, white or none	0 = do not eat, gluten free, white, or wholemeal bread 1 = wholegrain
Calcium intake	Frequency of dairy and dairy alternatives	5	> 5 d/week	None	0 = none 1 = 1 day/week 2 = 2 days/week 3 = 3 days/week 4 = 4 days/week 5 = 5 days or more
	Quantity of dairy/alternatives	5	> 4 serves/d	None	0 = 0 serves 1 = 1 serve or less 2 = 1.1–1.9 serves/d 3 = 2–2.9 serves/d 4 = 3–3.9 serves/d 5 = ≥ 4 serves/d
Iron intake	Frequency of lean red meat^^^	5	> 4 d/week	None	0 = 0 days or none **1 = 1–1.9 days**^¥^ 2 = 2–2.9 days 4 = 3–3.9 days 5 = > 4 days
	Quantity of lean red meat	5	> 4 serves/week	None	0 = 0 serves 1 = 1 serve or less 2 = 1.1–1.9 serves 3 = 2–2.9 serves 4 = 3–3.9 serves 5 = > 4 serves
					0 = 0 serves 0 = 1 serve or less 0 = 1.1–1.9 serves 0 = 2–2.9 serves 0 = 3–3.9 serves 1 = 4–4.9 serves 2 = > 5 serves
Sub-score		35			
Dietary habits					
	Regular intake over the day	2	‘Rarely’ or ‘never’ skip > 1 main meal on a weekly basis	‘Always’ or ‘usually’ skip > 1 main meal on a weekly basis	2 = Rarely/never skip main meal(s) 1 = sometimes skip main meal 0 = usually/always skip main meal
	Balanced food intake	2	‘Rarely’ or ‘never’ avoid > 1 core food group on a weekly basis	‘Always’ or ‘usually’ avoid > 1 core food group on a weekly basis	2 = Rarely/never avoid 1 or more core food group(s) 1 = sometimes avoid 0 = usually/always avoid one or more food group(s)
	Eat before training	2	‘Always’ or ‘usually’	‘Rarely’ or ‘never’	2 = always/usually 1 = sometimes 0 = rarely/never
	Eat after training	2	Always’ or ‘usually’	Rarely’ or ‘never’	2 = always/usually 1 = sometimes 0 = rarely/never
	Hydration	2	Water consumed as primary fluid before, during and/or after exercise. Water selected as a fluid choice on > 2 occasions (i.e., pre, during and/or post training)	Water not selected for any training occasions (i.e., pre, during and post training)	2 = water selected on 2 or more training options 1 = water selected on 1 training option 0 = water is not selected as a pre/during or post- training option
Sub-score		10			
Total score (H > 90; M:66–89; L < 65)		125			
